# Gliomatosis Peritonei and Its Relation to Teratoma: Role of Imaging and Histological Aspects

**DOI:** 10.7759/cureus.28849

**Published:** 2022-09-06

**Authors:** Tarang Patel, Virendrakumar Meena

**Affiliations:** 1 Pathology, All India Institute of Medical Sciences (AIIMS), Rajkot, IND; 2 Radiology, Geetanjali Medical College & Hospital, Udaipur, IND

**Keywords:** growing teratoma syndrome, teratoma, punctate fatty component, immature neuroepithelium, peritoneal glial implant

## Abstract

Gliomatosis peritonei (GP) is a rare disease, usually associated with immature ovarian teratoma. GP may be rarely associated with mature ovarian teratoma. GP is composed of mature glial tissue elements, which histopathological examination can further confirm. Benign glial implants usually involve the omentum, peritoneum and lymph nodes. Many benign and malignant peritoneal diseases may mimic GP on clinical examination. GP may be confused with peritoneal carcinomatosis on computed tomography (CT) scan. A microscopic examination from peritoneal mass biopsy helps to rule out differential diagnosis. GP consists of mature glial tissue and is regarded as grade 0 according to the WHO grading of immature teratoma (IT). GP corresponds to a good prognosis with occasional cases showing malignant evolution.

## Introduction and background

Gliomatosis peritonei (GP) is identified by multiple nodules affecting the omentum and peritoneum, and it shows mature glial tissue on histological examination. GP may be associated with immature or mature ovarian teratoma [1-3]. Cases have been published regarding the association of GP with hepatic teratoma, gastric teratoma and even immature endometrial teratoma [4-6]. Two theories prevail regarding the aetiology of GP. According to the first theory, GP results from rupture of ovarian teratoma capsule or angio-lymphatic tumour spread [7]. However, the second concept suggests that foci of peritoneal glial tissue arise autonomously from Mullerian stem cells under favourable peritoneal conditions [8]. CT imaging is helpful for the diagnosis and follow-up of immature teratoma (IT) and peritoneal glial implants. Surgery is performed in GP associated with teratoma, whereas chemotherapy is recommended in the case of IT [9].

## Review

Clinical and radiological features

IT clinically presents as a large pelvic mass, primarily turning up in the first three decades. Serum tumour markers like alpha-fetoprotein (AFP) are within normal limits [10]. On CT, IT shows some characteristic features. Mature teratoma (MT) are predominantly cystic tumours containing solid areas with large foci of fatty tissue that can be identified in radiology.

**Figure 1:**
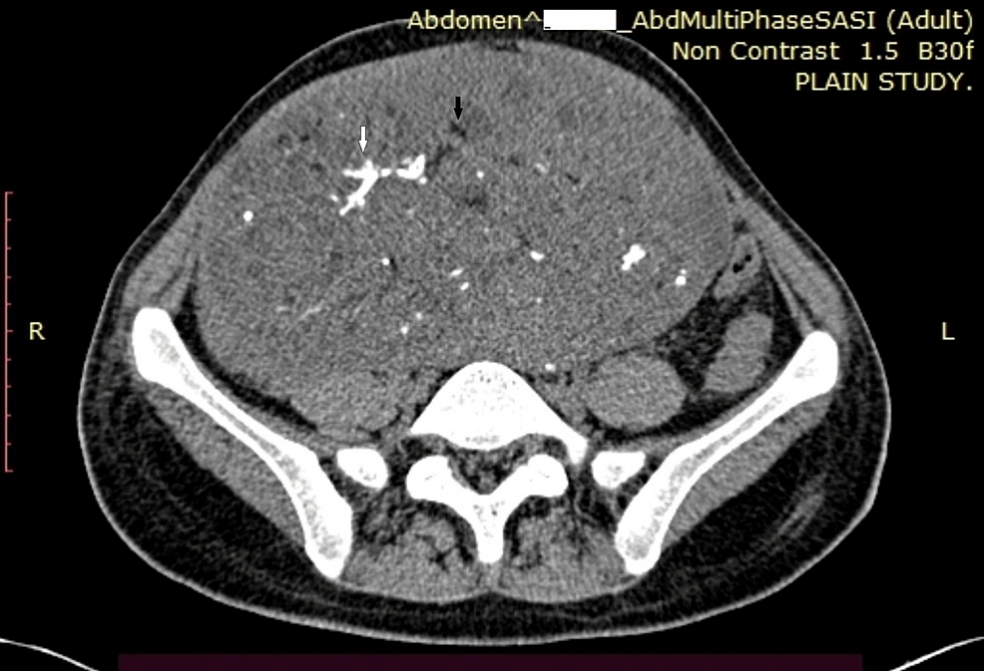
CT scan of immature teratoma ovary (Source: own photo) Axial computed tomography image shows a mass lesion in the pelvis with scattered fat (black arrow) and calcific foci (white arrow)

MT is usually well encapsulated with regular margins and foci of bony tissue within cystic elements. Compared to MT, IT is larger (14-25 cm) and shows predominantly solid components. IT usually presents with capsule perforation and irregular margins on a CT scan. Foci of punctate fatty tissue and scattered calcifications in a solid ovarian mass on CT scan discriminate IT from MT (Figure 1).

The combination of positron emission tomography (PET) and CT gives the additional benefit of providing morphologic and functional details in the case of IT [11].

**Figure 2:**
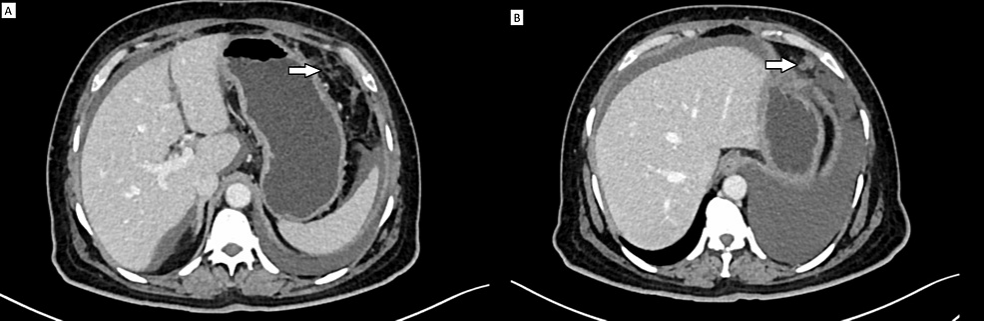
CT scan images of gliomatosis peritonei (Source: own photo) Post-contrast computed tomography (CT) axial section shows omental and mesenteric deposits in form of A) subtle fat stranding (arrow) and B) nodule (arrow)

GP on CT scan can present as multiple nodules involving the peritoneum and omentum with free fluid in the peritoneal cavity. The size of the peritoneal nodule varies from 0.3 to 1.2 cm in diameter [12]. It is challenging to differentiate peritoneal metastasis from GP on radio-imaging alone. Glial nodules are usually small in size without any adipose tissue component, making it impossible to separate tuberculous peritonitis or peritoneal carcinomatosis from glial nodules on imaging (Figures 2A-2B).

Radiology provides details concerning tumour staging and further surgery planning [13].

Gross and morphology

Intraoperatively GP may mimic peritoneal carcinomatosis. Grossly, GP presents multiple nodules having size ranging from 1 to 10 mm in diameter. They are small, greyish and well-circumscribed involving parietal and visceral peritoneum and omentum. Grossly it is challenging to differentiate GP from tuberculosis. IT presents as a large ovarian mass having an irregular outer surface. Tumour is mostly unilateral with cut surface showing mainly solid areas with focal cystic regions along with necrosis and haemorrhage [9,10].

**Figure 3:**
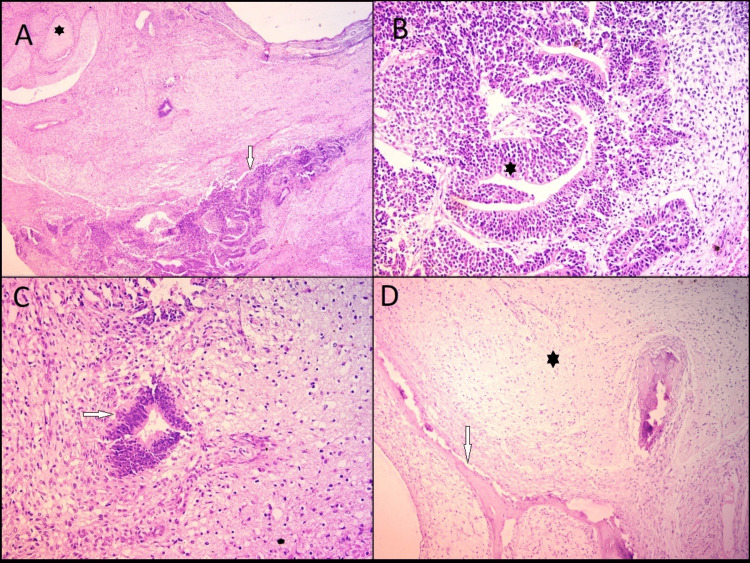
Immature teratoma ovary (Source: own photo) A) Section shows primitive neuroepithelium (arrow) embedded in the fibrillary background (H&E; 4×). B) Immature component in the form of primitive neural tubules (star) (H&E; 20×). C) Section shows foci of the immature neuroepithelium (arrow) in the background of mature glial tissue (H&E; 20×). D) Low-power view showing mature components in the form of mature brain tissue (star) and mature bony tissue (arrow) (H&E; 10×).

Microscopically, IT is defined by immature tissue components, mainly immature neuroepithelium in the form of rosettes and primitive neuroectodermal tissue in the background of mature teratomatous elements. Histology shows mitotically active primitive cells with dark hyperchromatic nuclei and glial tissue foci in the backdrop (Figures 3A-3D).

IT may present with immature mesodermal tissue in the form of immature cartilage. Very rarely, IT may show immature endodermal tissue. The case of IT should not show components of a yolk sac tumour; otherwise, the case will be labelled as a mixed germ cell tumour. If a histological picture of IT and serum AFP levels are raised, further additional sampling must be carried out to rule out any component of the yolk sac tumour [9,10].

**Figure 4:**
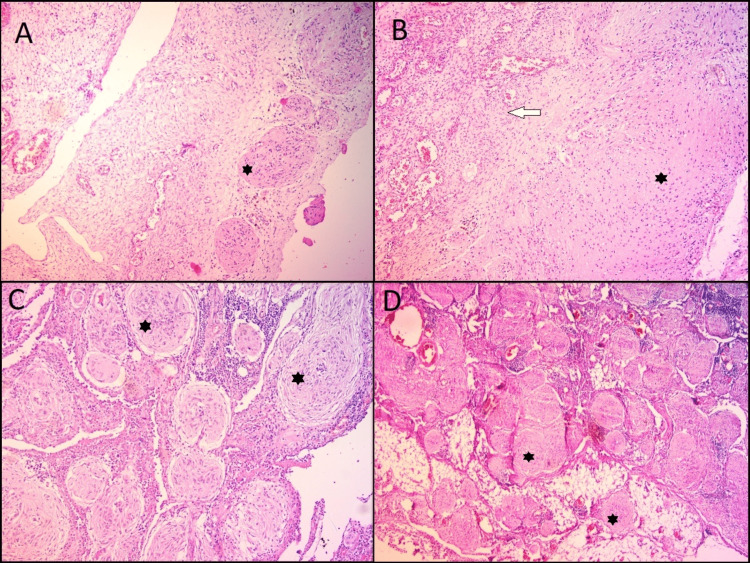
Gliomatosis peritonei histopathology (Source: own photo) A) Ovarian surface multiple superficial nodules of mature glial tissue (star) (H&E; 10×). B) Metaplastic glial elements (star) with diffuse arrangement on ovarian surface in a case of immature teratoma grade 1. Underlying ovarian stroma is evident (arrow) (H&E; 10×). C) Omentum shows multiple glial nodules (star) with reactive inflammatory stroma (H&E; 10×). D) Peritoneum showing gliomatosis in the form of multiple tiny glial nodules (star) infiltrating fibrolipomatous stroma (H&E; 4×).

According to WHO classification of Female Genital Tumours, 5th Edition; grading is decided by examining immature neuroepithelium under a low-power microscopic view (40×). If the aggregated amount of neuroepithelium is present in one or less than one 40× field, it is grade 1; if present in one to three 40× fields, it is grade 2 and if present in more than three 40× fields, it is classified as grade 3 IT. Here, 40× power is equivalent to a 4.5 mm diameter view. Interestingly, pure GP is regarded as a grade 0 (zero) mature component. It shows multiple nodules of mature glial tissue beneath the surface [10] (Figures 4A-4D).

On immunohistochemistry, GP cells show characteristic positivity with SOX2 because SOX2 is necessary for preserving stem cell pluripotency and induction of neural cells origin from stem cells [14].

Genetic profile

Best et al. presented molecular studies in patients of ovarian mass diagnosed as IT with GP. Results showed that genetic changes in both lesions were mutually exclusive, indicating that neoplasms of IT and GP are genetically discrete. So, both are independent tumours, and GP does not represent tumour metastasis or tumour recurrence from IT mass [15]. Another study by Ferguson et al. showed heterozygosity at different microsatellite loci and homozygosity at the identical microsatellite loci of cases of the peritoneal glial nodule and ovarian IT mass, respectively. So, this leads to an impression that GP arises from pluripotent stem cells of Mullerian origin and is not related to primary ovarian teratoma [1].

Differential diagnosis

Growing teratoma syndrome (GTS) is a distinct entity characterised by a growing mass of MT in a known case of malignant germ cell tumour undergoing or having completed chemotherapy. GTS may contain mature tissue from any of three lineages, whilst GP shows only mature glial tissue. Unlike GTS, GP is not associated with chemotherapy treatment of malignant germ cell tumours [16].

In some cases of ovarian teratoma, predominantly MT components are seen. If clinically, radiologically and grossly suspicious of immature or malignant nature, then extensive sampling should be conducted to detect any immature neuroepithelium and also need for accurate grading of the immature component. Peritoneal carcinomatosis and tuberculous peritonitis can be easily differentiated from glial nodules on histology [9,10].

Prognosis and predictive factors

The patient is staged according to TNM staging of the ovary, fallopian tube and primary peritoneal carcinoma. Although GP is considered a stage III disease, its behaviour is mainly observed as benign [10].

GP is considered a grade 0 (zero), so it usually bears an overall favourable prognosis and is treated conservatively accordingly [13]. As GP lesion is mostly widespread, extensive surgical excision is indispensable. However, luckily, any residual lesion of GP is asymptomatic and requires no further action, which may vanish over a long period of time [17]. Treatment is not decided based on metastatic glial tissue component but on the grade of primary ovarian IT, as long as the glial tissue has been extensively sampled and contains only mature tissue [18]. Although, if the peritoneal glial component is immature, it is considered a metastatic teratoma and treated accordingly [13]. At present, due to the rarity of this lesion, data are scarce regarding how long these cases should be pursued for follow-up.

## Conclusions

GP is an uncommon disease usually associated with IT of the ovary. In the case of ovarian teratoma, radio-imaging may help predict the immature or malignant nature of teratoma based on the identification of speckles of fatty tissue. In such suspected cases of IT, peritoneal and omental lesions should be searched, if any and should be excised along with ovarian mass to be sent for histopathological examination.

Grossly, throughout sampling should be carried out from ovarian mass to detect any immature neuroepithelium or other immature mesodermal components. A combined clinical, radiological and histopathological diagnostic approach is required to confirm the GP associated with IT and to differentiate it from its mimickers. It is also vital to acknowledge the benign mature nature of GP in most cases which helps to avert any avoidable surgical procedure in young patients.
